# Design and implementation of a hybrid cloud system for large-scale human genomic research

**DOI:** 10.1038/s41439-023-00231-2

**Published:** 2023-02-08

**Authors:** Masao Nagasaki, Yayoi Sekiya, Akihiro Asakura, Ryo Teraoka, Ryoko Otokozawa, Hiroki Hashimoto, Takahisa Kawaguchi, Keiichiro Fukazawa, Yuichi Inadomi, Ken T. Murata, Yasuyuki Ohkawa, Izumi Yamaguchi, Takamichi Mizuhara, Katsushi Tokunaga, Yuji Sekiya, Toshihiro Hanawa, Ryo Yamada, Fumihiko Matsuda

**Affiliations:** 1 Human Biosciences Unit for the Top Global Course Center for the Promotion of Interdisciplinary Education and Research (CPIER), Kyoto University, Kyoto, Japan; 2 Center for Genomic Medicine, Graduate School of Medicine, Kyoto University, Kyoto, Japan; 3 Academic Center for Computing and Media Studies, Kyoto University, Kyoto, Japan; 4 ICT Testbed Research and Development Promotion Center National Institute of Information and Communications Technology (NICT), Tokyo, Japan; 5 Division of Transcriptomics, Medical Institute of Bioregulation, Kyushu University, Fukuoka, Japan; 6 CLEALINK TECHNOLOGY Co., Ltd, Kyoto, Japan; 7 Genome Medical Science Project, National Center for Global Health and Medicine, Tokyo, Japan; 8 Department of Human Genetics, Graduate School of Medicine, The University of Tokyo, Tokyo, Japan; 9 Information Technology Center, The University of Tokyo, Chiba, Japan

**Keywords:** Data processing, Genetics research, Genomic analysis

## Abstract

In the field of genomic medical research, the amount of large-scale information continues to increase due to advances in measurement technologies, such as high-performance sequencing and spatial omics, as well as the progress made in genomic cohort studies involving more than one million individuals. Therefore, researchers require more computational resources to analyze this information. Here, we introduce a hybrid cloud system consisting of an on-premise supercomputer, science cloud, and public cloud at the Kyoto University Center for Genomic Medicine in Japan as a solution. This system can flexibly handle various heterogeneous computational resource-demanding bioinformatics tools while scaling the computational capacity. In the hybrid cloud system, we demonstrate the way to properly perform joint genotyping of whole-genome sequencing data for a large population of 11,238, which can be a bottleneck in sequencing data analysis. This system can be one of the reference implementations when dealing with large amounts of genomic medical data in research centers and organizations.

## Genomics: Designing and deploying a hybrid cloud computing system

Researchers in Japan have set up a hybrid cloud computing system for genomic analysis. A team headed by Masao Nagasaki of Kyoto University has designed and developed a scalable bioinformatics system and demonstrated its effectiveness by analyzing over 11,000 human genome sequences. The system combines local computing resources with supercomputers at Japanese universities and cloud computing from Amazon Web Services. The analysis pipelines were designed to be distributed across multiple sites while also ensuring reproducibility. Computing resources can be added as needed, for example, nodes based on graphics processing unit could be added for workflows that rely on deep learning, such as analysis of pathology images. This hybrid cloud computing platform not only provides a tool for researchers in Japan but also serves as a reference point for designing similar systems in other institutes or countries.

## Introduction

In recent years, whole-genome sequencing analysis has become increasingly commoditized, and a single organization or laboratory handles large amounts of sequencing information daily.

In addition, large-scale data are registered in public databases, enabling researchers in genomic medicine to access and utilize this information. For example, the UK Biobank^1^, a prospective genomic cohort study, provides more than 400,000 exome and 50,000 pieces of whole-genome sequencing data on a public cloud, Amazon Web Services (AWS: https://www.ukbiobank.ac.uk/enable-your-research/research-analysis-platform). The National Center for Biotechnology Information (NCBI) is a publicly available repository of high-throughput sequencing data^2^ with more than 36 petabytes through public clouds, such as the Google Cloud Platform (GCP) and AWS (https://www.ncbi.nlm.nih.gov/sra/docs/sra-cloud/). One solution is to set up sufficient computing resources on-premise (in-house) within a laboratory or organization. However, if the computational resources increase, achieving both setup and/or maintenance costs is usually difficult. In contrast, low-computing resources make it difficult to handle large-scale data analyses within a research period. Furthermore, various data analysis steps require different memories and CPUs; thus, the total number of computing resources and components change daily. Recently, in the United States, the AnVIL (the National Institutes of Health National Human Genome Research Institute (NHGRI) Genomic Data Science Analysis, Virtualization, and Informatics Lab-space) has been promoting a cloud platform running on the GCP designed to manage and store large-scale genomics to enable population-scale analysis (https://anvilproject.org/). As an international collaborative effort, researchers at the International Cancer Genome Consortium developed a unified interface for searching and accessing data to authorized users from a commercial cloud, AWS, and an academic cloud, the Cancer Genome Collaboratory (https://dcc.icgc.org/icgc-in-the-cloud).

As one of the solutions, we describe the design and implementation of a hybrid cloud system consisting of an on-premise supercomputer, science cloud, and public cloud at our center, the Kyoto University Center for Genomic Medicine, in Japan. It can flexibly handle various heterogeneous computational resource-demanding bioinformatics tools while scaling the computational capacity. Our center handles a prospective genomic cohort study of 10,000 participants in Japan^3^ and organizes a Japanese government-authorized registry of rare disease repositories in Japan (RADDAR-J: http://raddarj.org/en/)^4^. Whole-genome data analyses of thousands of individuals are becoming common in human genome analysis in one laboratory or organization. Our center has also been storing and analyzing whole-genome sequences of more than 10,000 samples, including the above cohort participants and rare diseases.

For the whole-genome sequencing analysis of each individual, bioinformatics tools with a CPU^5,6^, graphics processing unit (https://www.parabricks.com/), field-programmable gate array (FPGA) technology^7^, and cloud-based solutions^8,9^ exist^10^. However, a particular bottleneck usually occurs after an individual data analysis, joint-genotyping analysis, in which genotype information from all samples (e.g., 1000 or 10,000 individuals) is simultaneously accessed and analyzed to improve the accuracy and recall of individual genotyping results. If new samples are analyzed in the former individual data analysis step, the joint-genotyping operation needs to be reanalyzed from scratch.

For the 2535 samples from low-coverage WGS sequencing data in the 1000 Genomes project, Real-Time Genomics population callers were used to manage the joint-genotyping analysis on AWS^11^. For the 5297 samples from low-coverage WGS sequencing data (6X to 10X coverage) in the Cohorts for Heart and Aging Research in Genomic Epidemiology WGS freeze3 dataset, four joint-genotyping analyses, SNPTools, GATK-HaplotypeCaller Ver. 3, GATK-UnifiedGenotyper Ver. 3 and GotCloud^12^, were performed on their hybrid cloud system^13^. In the United States, the NHLBI Trans-Omics for the Precision Medicine (TOPMed) Program recently released 53,831 population panels from high-coverage WGS sequencing data using GotCloud (https://genome.sph.umich.edu/wiki/GotCloud)^14^. In the UK, the largest population panel is the 150,119 samples from high-coverage WGS sequencing data in the UK Biobank using GraphTyper Ver. 2^15,16^.

Based on this progress, to demonstrate the effectiveness of the hybrid cloud system, we describe the joint-genotyping operation of 11,238 WGS using GATK Ver. 4 on our system, addressing the different features and computational performance of each subsystem. The relationship between sample size and processing time of joint-genotyping operations is also discussed per the measured results on real datasets from 149 to 11,238 samples. Finally, the execution times for larger WGS datasets of 20,000, 30,000, 40,000, and 50,000 samples are estimated.

Depending on the institution and country, on-premise computing resources, supercomputing systems, public clouds, network environments, and ethical constraints must be considered in system design and implementation. Nevertheless, our hybrid cloud system provides a good starting point for reference.

## Materials and methods

### Hybrid cloud system

#### Overview of subsystems and network

**Fig. 1:**
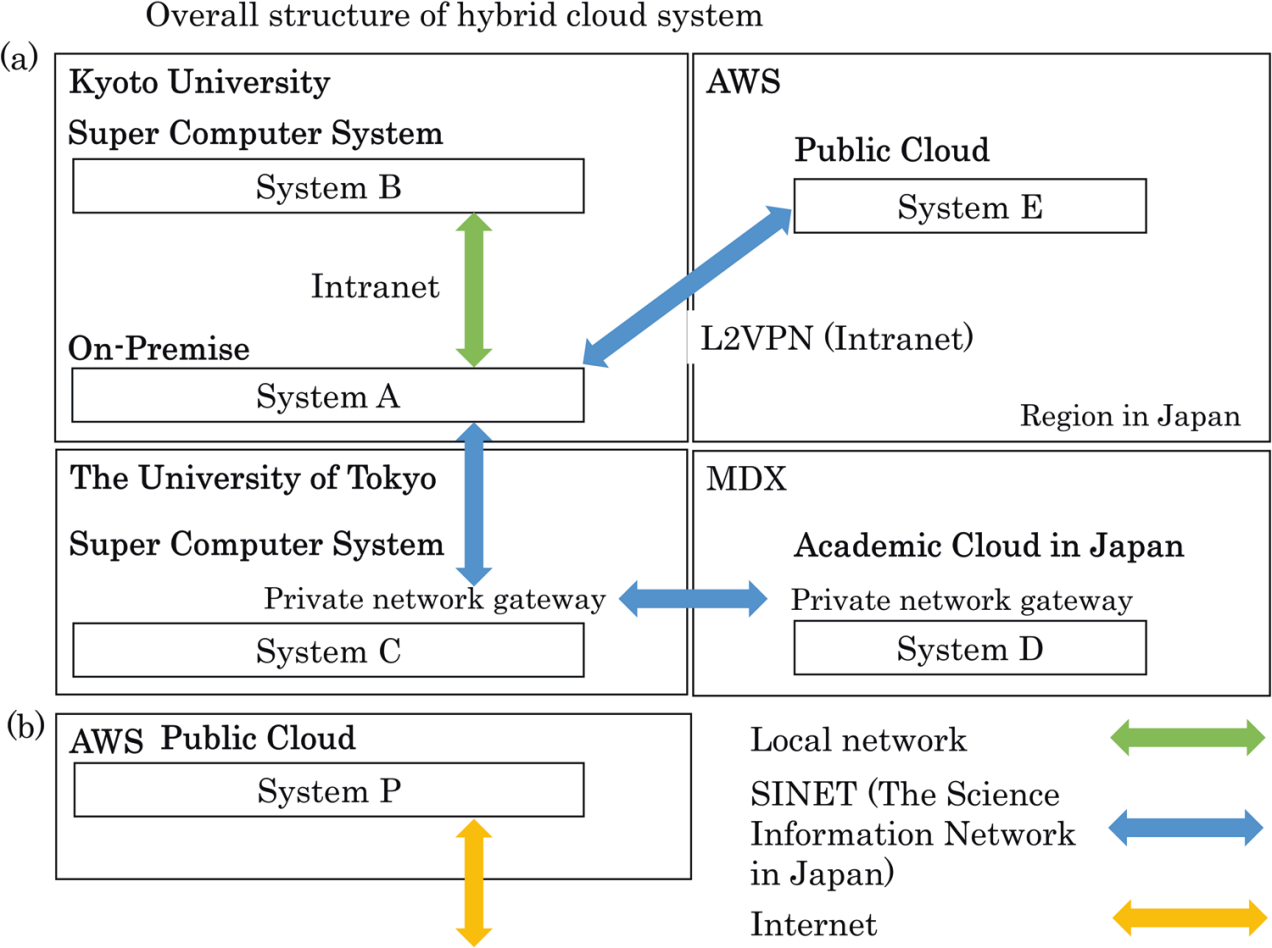
Overall structure of the hybrid cloud system. The double-headed arrows indicate different types of network connections. **a** Private system. Subsystems A–E are connected by the SINET network at 100–400 Gbps. **b** Public system in a public cloud. The network is independent of (**a**).

The overall structure and specifications of the hybrid cloud system at the Kyoto University Center for Genomic Medicine are shown in Fig. 1 and Table 1, respectively. The computer resources and storage consisted of on-premise (System A), a supercomputer system at Kyoto University (System B: https://www.iimc.kyoto-u.ac.jp/en/services/comp/supercomputer/), a supercomputer system at the University of Tokyo (System C: https://www.cc.u-tokyo.ac.jp/en/guide/hpc/obcx/), an academic cloud mdx (System D: https://mdx.jp/en/)^17^, and a public cloud, Amazon Web Services (AWS), in the region of Japan (System E). Systems A to E are connected via the Japanese academic network called the science information network (SINET: https://www.sinet.ad.jp/en)^18^. As an intranet system of our organization, System E is directly connected only to system A using SINET L2VPN with 10-GB bandwidth. From Apr/2022, SINET has been updated from version 5 (100 Gbps bandwidth connection) to version 6 (toward 400 Gbps). High-performance data transfer between subsystems is an essential factor in hybrid cloud systems. Our center prepares a high-speed data transfer tool, the HCP tool, between subsystems. The HCP tools are designed and implemented based on a high-performance and flexible protocol, HpFP, which is a packet-loss tolerance and thus has outstanding performances of data transfer on LFNs (long-fat networks)^19^, mobile networks^20^, and satellite communication networks^21^.

**Table 1:** Specifications of the hybrid cloud system at the Center for Genomic Medicine, Kyoto University, Japan.

Subsystem	Name	Specification	Size
System A	File System	GPFS ESS JBOD (5U84)	2.1 PB
	File System	NAS	3.6 PB
	Compute Nodes	Intel Xeon Haswell E7-8890 v3 (Haswell, 18 cores 2.5 GHz × 4)/512 GiB	Three nodes
	Compute Nodes	Intel Xeon Ivy Bridge E7-4880 v2 (Ivy Bridge, 15 cores 2.5 GHz × 4)/512 GiB	One node
	Compute Nodes	Intel Xeon E5-2698 v4 (Broadwell, 20 cores 2.2 GHz × 2)/8× Tesla P100 GPU/512GiB	One node
	Job Schedular	Slurm	
	Container	Singularity v3	
	Location	Center for Genomic Medicine, Kyoto University, Japan	
System B	System name	Laurel 2	
	File System	Lustre ExaScaler (SFA14K)	0.71(24) PB
	Compute Nodes	Intel Xeon E5-2694 v4 (Broadwell, 18 cores 2.1 GHz × 2)/128 GiB	17 (850) nodes
	Network	Between network gateways and SINET (100 Gbps) Between file systems and compute nodes (100 Gbps)	
	Job Schedular	PBS	
	Container	Singularity v3	
	Location	Academic Center for Computing and Media Studies, Kyoto University, Japan	
System C	System name	Oakbridge-CX (OBCX)	
	File System	Lustre ExaScaler (ES18KE × 2)	0.7 (12.4) PB
	Compute Nodes	Intel Xeon Platinum 8280 (CascadeLake, 28 cores 2.7 GHz × 2)/192 GiB	256 (1368) nodes
	Network	Between the private network gateway and SINET (10 Gbps) Between public network gateways to SINET (40 Gbps × 2) Between file systems and compute nodes (100 Gbps)	
	Job Schedular	Fujitsu Technical Computing Suite-	
	Container	Singularity v3	
	Location	Information Technology Center, the University of Tokyo, Japan	
System D	System name	mdx	
	File System	Lustre File Sytem (NVMe)	0.15 (1) PB
	File System	Lustre File Sytem (HDD)	0.3 (16.3) PB
	Compute Nodes	Virtualization environment with 608 cores and 1024 GiB in total. 76 cores (152 vCPUs) and 128 GiB are assigned to each compute node. The physical CPU is Intel Xeon Platinum 8368 (IceLake, 38 cores 2.4 GHz × 2).	8 (368) nodes
	Network	Between compute nodes and SINET (25 Gbps)	
	Job Schedular	Slurm	
	Container	Singularity v3	
	Location	Information Technology Center, the University of Tokyo, Japan	
System E/P	File System	FSx for Lustre	On-demand
	File System	EBS	On-demand
	File System	S3	On-demand
	Network	Between System A to AWS (AWS Direct Connect via SINET (10 Gbps))	
	Compute Nodes	AWS assigns requested instance if compute nodes are physically available. For our GATK joint-genotyping in System E, spot instance of r5.large (2 × vCPU (Intel Xeon Platinum 8000 3.1 GHz)/16 GiB)) was used (max 320 nodes). Available max resources as spot instance of vCPU and memory in AWS region in Japan are hpc6a.48xlarge (AMD EPYC Milan) with 192 vCPUs and x2iedn.32xlarge (Intel Xeon IceLake) 4096 GiB at 1/Jul/2022, respectively.	Spot
	Location	AWS region in Japan (Systems E and P are in different virtual private cloud)	

*PB* petabyte, *GiB* gibibyte.

The value outside the brackets indicates the value currently being rented, and the value inside the brackets indicates the total system.

System P is hosted in AWS as an independent network from other systems for public services, such as web and database services.

#### Storage

System A provides both online and offline storage solutions as a centralized information hub. As an online solution, System A consists of high latency and low-latency storage, a high-speed distributed parallel file system (General Parallel File System (GPFS), a total of 2.1 petabytes), and a network-attached system (NAS, a total of 3.6 petabytes). High-latency storage stores and operates information frequently accessed from on-premise computing nodes and transferred from/to other systems. Low-latency storage stores information that is accessed infrequently by users. For example, once a month, long periods of direct access are required by computational resources, for raw sequence data (fastq file: http://samtools.github.io/hts-specs/SAMv1.pdf). As an offline solution that stores information to archive for long periods (e.g., store data analysis pipelines and results used for a publication to ensure the reproducibility of a publication), general hard disk drives (HDDs) are used by temporally attaching them to a compute node in System A. These offline archived HDDs are maintained in a secure area with the power turned off.

Systems B, C, and D install a Lustre-based file system, a high-speed distributed parallel file system (https://www.lustre.org/). Of the 24, 12.4, and 17.3 petabytes in Systems B, C, and D, our center rented a total of 0.71, 0.7, and 0.45 petabytes, respectively. Storage in these regions is used for fast read/write access from their local compute nodes and as backups for critical partial data for disaster recovery.

Systems E and P are flexibly chosen from the high-speed storage, FSx Lustre, general storage, EBS, or near-line storage, S3, for each bioinformatics tool, depending on the latency needed. Most tools are processed by temporally attaching an encrypted EBS or S3 to compute nodes.

#### Container and workflow

A hybrid cloud system assumes that analysis pipelines should be distributed and processed among multiple sites, that is, Systems A to E. Therefore, creating an environment where the analysis pipelines among multiple sites can be unified as much as possible is essential; otherwise, reproducibility will be lost, as well as a different software version at another site and the cost of rewriting analysis pipelines to run on different sites. Therefore, our current hybrid cloud system uses the following solutions. All the computation nodes in Systems A–F use one of the containers, Singularity version 3 (https://sylabs.io/docs/). In advance, frequently used bioinformatics tools, such as bwa^22^, bcftools^23^, and samtools^23^, are downloaded from public image repositories or compiled and created in Singularity version 3 image format (SIF). The SIF image files were placed in the predefined directory path at multiple sites. This allowed us to reproduce and minimize the rewriting cost of pipelines at different sites. Most bioinformatic tools have been implemented and tested on Intel-based CPU architectures. Furthermore, some tools cannot even be compiled on other CPU architectures, such as, for example, using Intel-based CPU extension instructions in the tool to boost the calculation speed. Thus, our singularity images are compiled for the Intel architecture and do not work on other architectures, such as Fujitsu A64FX and AWS Graviton 2; in other words, our hybrid cloud system cannot use the EC2 instance Gravition2 in Systems E and F. As batch job systems, Slurm has been installed for Systems A, D, E, and P, which can be controlled as the administrator by our technical staff. Almost identical analysis pipelines can be executed among these systems. Slurm is an open-source, fault-tolerant job scheduling system and a highly scalable cluster management system for small to large Linux clusters (https://www.schedmd.com/). For Systems B and C, different batch job systems are preinstalled and serviced to users without administrative privileges, including researchers in our center. Therefore, the analysis pipelines still need to be manually rewritten for these batch job systems. AWS provides an AWS batch and a proprietary batch system. However, System E uses Slurm to avoid vendor locks (to ensure portability to other systems). For more information on the advanced use of containers and workflow description languages, for example, WDL (https://openwdl.org/) and CWL (https://www.commonwl.org/v1.2/), workflow engines, such as Nextflow^24^ and Cromwell (https://github.com/broadinstitute/cromwell), refer to the Practical Guide to Managing Large-Scale Human Genome Data Analysis^10^ or elsewhere.

#### Computational resources

In bioinformatics analysis, some tools require a long processing time, for example, more than one week, and large memory, such as 512 gibibytes (GiB). Therefore, to take advantage of a hybrid cloud system, it is practical to select appropriate computing resources by analyzing the resources required from each tool.

For System A, which our center manages, the maximum execution time of each process (Slurm batch job) is not set except for the scheduled maintenance. For System D, the virtualization system allows administrative privileges for the launched virtual compute nodes, and the maximum execution time of each process is not set. These settings allow us to evaluate and analyze the required computational resources of each bioinformatics tool by using a partial real dataset before large-scale data analysis in other systems. However, the maximum job running times in Systems B and C are limited to one week and two days, respectively. System E has no upper limit, mainly when an on-demand instance is selected in the AWS. Therefore, jobs that require a long running time can be performed on Systems A, D, and E. Systems A and D are the first choices, whereas if a strict deadline exists and only Systems A and D can process partial jobs until the deadline, then System E also processes the job. Additionally, jobs will also be processed on System E.

The available memory size of each computing node is 128, 192, 512, and 4096 GiB for Systems B/D, C, A, and E, respectively (ordered by memory size). Most bioinformatics analysis tools can process within the memory sizes provided by Systems B/D and C. The remaining analyses, which require more substantial memory, are performed on System A as the first choice. When that is still insufficient, the analyses are performed on System E. This strategy almost eliminates the problem of running out of memory for bioinformatics tools.

## Results

### Use case of analyzing 11,238 whole genomes on the hybrid cloud system

#### Overview of the whole-genome sequence data analysis

**Fig. 2:**
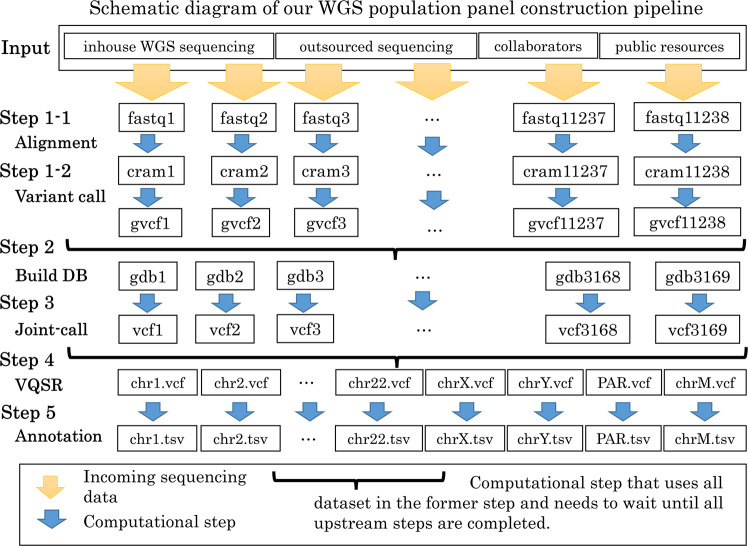
Schematic diagram of our WGS population panel construction pipeline. The diagram depicts the data processing flows of whole genome sequencing data. The orange arrow indicates the incoming sequencing data from various sources, and the blue arrow indicates the computational steps from Step 1 to Step 5.

Figure 2 shows a schematic diagram of our whole-genome sequence (WGS) pipeline from Step 1 to Step 5. Whole-genome analysis generally starts from an individual variant detection step (Step 1), in which the sequenced data, e.g., fastq, from each individual is aligned to an international reference assembly, e.g., GRCh37, GRCh38, or CHMv2^25^ (in our analysis, the same reference assembly, GRCH38DH, in the 1000 genomes project was used (ftp://ftp.1000genomes.ebi.ac.uk/vol1/ftp/technical/reference/GRCh38_reference_genome/)), and generates genotyping information, e.g., variant call format (VCF) or general VCF (gVCF)^26^, of the different bases and the same bases in gVCF from the used reference assembly with statistics for each base, e.g., strand bias and phasing information estimated from the sequenced reads. If a population-based analysis is not needed, Steps 2 to 4 can be skipped, and the VCF in Step 1 is directly used in Step 5.

In the next steps (Step 2 and Step 3), a joint-genotyping operation is usually applied to improve the precision and accuracy of variant detection and allow each statistical indicator of the genotyping information from different individuals to be easily compared. The operation takes many individuals’ information (gVCF files from 100 or 10,000 in the former step) as input, merges information, and generates files with genotyping information of multiple individuals (usually gVCF files with multiple individual data).

The last step of genotyping (Step 5) is to use a machine learning model, e.g., a Gaussian mixture model, by using the training set of high-confidence variants, e.g., known SNPs in public databases, assign a reliable score to each variant and generate VCF files.

Step 5 then applies biological annotations to VCFs in Step 1 or Step 4 to ease the interpretation of biological impacts of each variant for researchers, e.g., a loss-of-function variant or/and a reported variant in the GWAS catalog database^27^.

#### Step 1: Individual whole-genome alignment and genotyping

Our center stores the fastq files of whole-genome sequencing data from various sources: sequence data generated from in-house sequencers, obtained through outsourcing to sequencing companies, and shared through data sharing processes from public databases, e.g., sequence read archive (SRA)^2^ or NBDC Human databases (https://humandbs.biosciencedbc.jp/en/).

The data analysis in Step 1 for individual sequence data, fastq, is independent of other individual sequencing data. Therefore, the individual sequence data analysis in Step 1 can be performed immediately once the sequencing is complete (Input, Step 1-1 and Step 1-2 in Fig. 2). For the current fastq files in our center, each job in Step 1 can be completed within two days of all compute nodes with less than 128 GiB memory. Therefore, Step 1 can be performed in any System A to E. Normally, the analysis is performed on Systems A, B, or C using CPU-based software (Steps 1-1 and 1-2 in Table 2). The mean and median job processing times in Systems A, B, and C were not too different. Our center has allocated System A primarily because of its fixed annual cost. If the computational capacity in System A was occupied by including other users’ jobs, then the fastqs were transferred to Systems C and B and analyzed on these systems. When it is necessary to complete the analysis within a certain period, it can also be carried out by temporally allocating computing resources to System E.

**Table 2:** Summary information of the tools, computing resources and run time in each pipeline step.

Pipeline Step	Operation	Application	Location	Total job run time (hour)	Mean job run time (min)	Median job run time (min)	Total Job (job count)	Requested Resouce per Job (# the reason why does not use the location)
Step 1-1	Alignment	bwa ver. 0.7.17 Reference hs38DH.fa (hs38, ALT contigs, decoy contigs, and HLA genes)	System A	24735.4*	652.9	548.9	2273	20 cores/memory 32 Gb
			System B	1255.2	753.1	740.1	100	32 cores/memory 120 Gb
			System C	95797.4*	648.4	648.4	8865	System C allows only job assignment per compute node. 56 cores/memory 192 Gb
Step 1-2	Variant call	GATK ver. 4.1.4 HaplotypeCaller	System C	181964.2*	971.5	1122.6	11,238	System C allows only job assignment per compute node. 56 cores/memory 192 Gb
Step 2	Genomic DB import	GATK ver. 4.1.4 GenomicDBImport	System A	56,202.6	1064.1	1044.0	3169	Memory 18 Gb
Step 3	Joint-Genotyping	GATK ver. 4.1.4 GenotypeGVCFs	System A	63638.1*	18,445.8	19,352.0	207	Memory 16 Gb
			System B	–	–	–	–	#Most of the jobs cannot complete within max running time (2 days).
			System C	–	–	–	–	#Cannot process jobs with the overload of I/O access.
			System D	56242.7*	5709.9	5834.1	591	memory 16 Gb (chr1) memory 10 Gb (chr3,9)
			System E	45630.9*	1154.7	1377.2	2371	Two cores/memory 16 Gb (r5large)
Step 4	Variant quality score recalibration (VQSR)	GATK ver. 4.1.4 VariantRecalibrator and ApplyVQSR	System A	872.0	5813.4	82.2	28	VariantRecalibrator (INDEL/SNP) memory 32 G/288 Gb ApplyVQSR memory 128 Gb
Step 5-1	Annotation	SNPEff Ver.4.3i	System A	10.9	25.2	25.5	26	Memory 12 Gb
Step 5-2	Annotation	VEP API Ver.106 DB Ver.105	System A	797.6	1840.6	1791.7	26	Memory 16 Gb

The total jobs, the run time (mean/median/total) and resource allocation of computing resources are summarized in each analysis step.

*Total job run time was estimated from the mean job run time in the logged jobs.

As an independent high-speed data processing solution in Step 1, the FPGA genotyping system DRAGEN™^28^, is installed in System A. The FPGA system allows the processing of an individual fastq with 40x coverage of the human reference assembly within 30 min. When a very fast Step 1 analysis is required for a small dataset as a quality control purpose in our center, e.g., one to 100 samples, it is sometimes operated on the FPGA system as an independent dataflow from the former primary CPU-based solution. In AWS, the same FPGA genotyping system is already serviced in several regions in the United States, Germany, Australia, and Ireland. However, it is still not installed in any other region, including Japan, as of July 2022. The implementation of alignment and variant calls in DRAGEN™ is different from the former primary CPU-based solution. Thus, if the cram and gVCF are created from the fastq of a sample by DRAGEN™, the fastq of the sample is always reanalyzed by the CPU-based solution. After processing 11,238 samples, the total sizes of the cram and gVCF were 123.5 TB and 186.8 TB, respectively (Step 1-1 and Step 1-2 in Table 3).

**Table 3:** Summary information of storage allocation in each pipeline step.

Pipeline Step	Operation	Input format	Output format	Total file size (Tb)	Mean size (Gb)	Median size (Gb)	Total file numbers	Note
Input				480.2	21.9	38.5	22,476	Consist of two files for each sample, i.e, paired-end protocol.
Step 1-1	Alignment	fastq	cram	240.4	21.9	19.4	11,238	Not include crai index file.
Step 1-2	Variant call	cram	gvcf	123.5	0.4	0.4	288,290	Not include tabix index file.
Step 2	Genomic DB import	gvcf	gdb	186.8	60.4	67.7	28,269,278	The mean and median size are the total file size per interval (in total 3169 interval dataset.)
Step 3	Joint-genotyping	gdb	vcf	5.8	226.9	204	26	Chr1-22/X/Y/PAR/M Not include tabix index file.
Step 4	Variant quality score calculation	vcf	vcf	10.0	395.6	355.3	26	Chr1-22/X/Y/PAR/M Not include tabix index file.
Step 5	Annotation	vcf	tsv	0.1	3.3	3.3	27	
			Total	1046.8			28,591,361	

The file size (mean/median/total) and total file numbers are summarized in each analysis step.

#### Step 2: Variant database construction

For the population joint-calling tools, the commands GenomicDBImport and GenotypeGVCF of the Genome Analysis Tool Kit (GATK)^5^ from the Broad Institute or the command GLnexus from DNANexus^29^ are usually used. In our center, to conduct whole-genome analyses of 11,238 individuals, we used the former tool according to the best practice of GATK version 4.

The GenomicDBImport operation in Step 2 constructs databases in GenomicDB format, such as a specialized TileDB (https://tiledb.com/) for genomics applications, e.g., VCF parsing and INFO field annotation calculation. TileDB is a database format for efficiently representing sparse data, such as genotype data from individuals, because most positions are the same as reference bases to the international reference assembly. In the GenomicDBImport operation, chromosomes are divided into regions, and the GenomicDB database (interval database) is constructed for each region.

In our center, chromosomes 1–22, X, Y, and M are divided into 3169 regions and processed (Fig. 2 in Step 2). In particular, the processes can be performed independently for each divided region, and the GenomicDBImport operation for each region can be performed in parallel (Fig. 2 in Step 3) between Systems A to E. As the construction of an interval database requires the gVCFs of all target individuals, i.e., 11,238 files, our center uses System A, which collects all gVCF files in advance to the master data repository after the Step 1 process is completed (Step 2 in Table 2). When creating one interval database, 4506 to 9314 files were created per region (the mean and median total sizes of one region were 60.4 and 67.7 GB, respectively). A total of 11,238 samples were processed to build 3169 gdb, totaling 28,269,278 files of 186.8 TB (Step 2 in Table 3).

#### Step 3: Population joint-genotyping analysis

The next GenotypeGVCF operation takes an interval database and generates the genotype information of all 11,238 samples in the chromosomal interval as a gVCF file. In total, 3169 regions must be processed (Step 3 in Fig. 2). These processes can also be performed independently for each interval database; thus, ideally, the GenotypeGVCF operation can be performed in any of Systems A–E.

Unfortunately, a practical problem still occurs when the interval database files accessed by each process are different. Suppose all processes are operated on one distributed file system, as in the worst-case scenario; approximately 30 million files would be accessed simultaneously from 3169 processes. Therefore, even in high-performance parallel distributed file systems (Lustre file systems and GPFS), these processes affect the processing performance. In our case, with the Lustre file system, System B was constrained by the administrator to limit the number of concurrent file access operations to less than 50,000 per second (before the limitation, the access of parallel jobs reached more than 200,000 per second). Subsequently, all processes in System B were suspended and rescheduled to other systems. Therefore, we limited the maximum number of concurrent jobs in Systems A and D. In System E, by considering the above features of the joint-genotyping operation, we attempted to minimize the bottleneck and scale for concurrent processes by implementing the following solutions. While the concurrent jobs in Systems A and D were limited, the computational times in System A (the mean and median are 18,445.8 and 19,352.0 min) and System D (5709.9 and 5834.1 min) were clearly slower than in System E (1154.7 and 1377.2 min), as seen in Step 3 in Table 2. The breakdown of the detailed usage of each subsystem for the total 3169 joint-genotyping processes is summarized in Supplementary Table 1a.

For the analysis in System E, in advance, a single compressed file (in tgz format) is created for each interval database in System A, and the compressed file is transferred from System A to the compute node in System E, i.e., the EC2 instance. This eliminated one possible bottleneck problem caused by transferring many files. Each computing node directly expands 4506 to 9314 files into the attached local file system, in this case EBS, to the computing node. By assigning each local file system that differs from the other computing nodes, we attempt to minimize the network dependencies among the compute nodes. This eliminated the other possible bottleneck problem caused by accessing a large number of files from many computing nodes.

Furthermore, to minimize the cost of computing nodes, we used the instances of the spot plan in the AWS. Compared to the on-demand plan, the computing node has one disadvantage: the node might be terminated by requests from other cloud users, mainly from the on-demand plan. Therefore, if the job of a spot instance is forced to terminate before completing the job, our custom script resumes restarting from the joint-genotyping operation by skipping the joint-genotyping regions already processed in the former job. For 2371 regions processed in System E, 0 (no resume), 1, 2, 3, 4, and 5 were 1403, 720, 212, 23, 12, and 1, respectively (Supplementary Table 1b). The maximum resume count was five, and 3637 jobs were required for 2371 regions in System E. To process 11,238 samples, r5.large virtual computing nodes (two vCPUs from Intel Xeon Platinum 8000 3.1 GHz and 16 GB memory; $0.0366/h per node) were selected as the Slurm client nodes. The selection of instances would be changed according to the total number of joint-genotyping samples. After processing 3169 regions, the total size of vcf was 5.8 TB (Step 3 in Table 3).

#### Step 4: Calculate and assign variant quality scores

For the downstream analysis of Step 3, the new variant quality score called the VQSLOD (for variant quality score log-odds) is calculated with the VariantRecalibrator operation. The score is usually used as an essential measure to distinguish reliable variants from unreliable variants for the downstream ApplyVQSR operation. To calculate VQSLOD, all VCF files in Step 3 are needed. In our center, the calculated VCFs and gVCFs at other systems are always gathered in the master storage of System A. In addition, the ApplyVQSR operation cannot be split into independent processes. Thus, both the VariantRecalibrator and ApplyVQSR operations are processed in System A. After processing 26 regions, the total size of vcf was 10 TB (Step 4 in Table 3).

#### Step 5: Biological annotation

As the basic annotations, the major annotation tools, VEP^30^ and SnpEff^31^, are used in our center on System A with the role of the master dataset (Step 5 in Fig. 2). Four annotation tasks took more than two days for the 11,286 population panel by still splitting jobs by each chromosome region. After processing, the total annotation file size was 0.1 TB (Step 5 in Table 3).

## Discussion

**Fig. 3:**
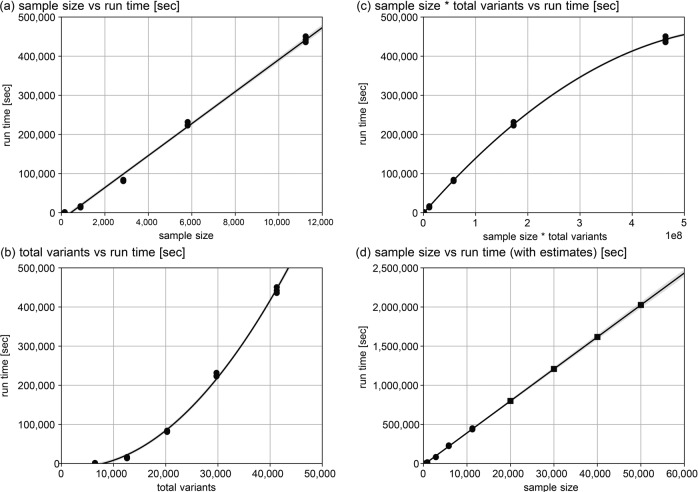
The performance evaluations and estimations for the joint-genotyping operation. The relationship between running time and (**a**) sample size, (**b**) total variants in the chromosomal region, and (**c**) multiples of (**a**) and (**b**) measured three times for 149, 878, 2847, 5809 and 11,238 samples. In (**a**) to (**d**), the circles are the measured results on System D. The line in (**a**) is the result of linear regression, and the gray region is within one sigma interval. The curves in (**b**) and (**c**) are the polynomial regression of degree two, and the gray region is within one sigma interval. The rectangles in (**d**) are the estimated total run time on System D for sample sizes 20,000, 30,000, 40,000, and 50,000 to the same chromosomal region from the linear regressed result in (**a**).

We introduced the hybrid cloud system at our center as a reference implementation for adaptively handling the increasing large-scale information in genomic medical research. In the hybrid cloud system, we demonstrated the workflow of the whole-genome analyses of 11,238 individuals. Apart from this whole-genome analysis, adding more GPU-based nodes to the current hybrid cloud system can also be applied to deep learning tools that are necessary for genomic medical research, e.g., spatial-omics images or pathological diagnostics. One advantage of the hybrid cloud system to one sole system (e.g., one supercomputer system) is the flexibility to change the computing resource proportion of each subsystem (i.e., Systems A to E and P in our center) on a daily basis, considering the system requirements of mainly processing bioinformatics tools, the data needed to process, and the technical progress of each subsystem (e.g., usually a supercomputer system is replaced every five to six years in Japan). Especially in our case, System C allows large computational resource assignments in Step 1. In Step 3, System E resolves the I/O bottleneck problem, which was faced in Systems A, B, and D. Additionally, System A allows the role to gather and store all individual sample data in Step 1 and store population data in Steps 4 and 5 as the master dataset. System B serves as the backup storage for System A. To estimate the relationship between the sample size and execution time of the joint-calling operation in Step 3, among chromosomal 3169 regions, we selected the chromosomal region with the median gdb size in Step 3 (chr6: 74, 371, 373–75, 371, 372) and measured the execution time for the sample size, 149, 878, 2847, 5809 and 11,238 with 6431, 12,571, 20,259, 29,729, and 41,279 variants on System D without running multiple jobs in the same computational node (to avoid I/O bottleneck). Figure 3 displays the plot of the relationship between running time and (a) sample size, (b) total variants in the chromosomal region, and (c) multiples of (a) and (b). The result implies that the computational time has a linear relationship with the sample size of the chromosomal region. The estimate also allows us to calculate the required computational time for the same chromosomal region with 20,000, 30,000, 40,000, and 50,000 total samples at ~0.8, 1.2, 1.6, and 2.0 million seconds on System D, respectively (Fig. 3d and Supplementary Table 2).

In general, the size of the storage requirements inevitably increases yearly due to the increase in the amount to be analyzed, e.g., sequencing information, and the demand to keep the results of analyses obtained in research activities for reproducibility. Currently, the public cloud is advantageous as it bursts computing resources for temporally needed jobs in the case of the joint-genotyping operation in Step 3 and for database and web services that require full-time services. For example, Systems A to D have scheduled maintenance, e.g., once a month in System C, and System B needs to stop for three months to migrate the contents to the new supercomputer system in the current Lustre file system. However, our center considers that it is not yet cost-effective to migrate our storage systems to public clouds, mainly provided on the premises and supercomputing systems. If the cost issue is improved in the future, we may increase the weight to shift our storage system to public clouds with disaster recovery and encryption capabilities.

### Supplementary information

Supplementary Table 1

Supplementary Table 2
